# Network analysis of aging acceleration reveals systematic properties of 11 types of cancers

**DOI:** 10.1002/2211-5463.12679

**Published:** 2019-06-24

**Authors:** Xiaoqiong Xia, Mengyu Zhou, Hao Yan, Sijia Li, Xianzheng Sha, Yin Wang

**Affiliations:** ^1^ Department of Biomedical Engineering School of Fundamental Sciences China Medical University Shenyang China; ^2^ Tumor Etiology and Screening Department of Cancer Institute and General Surgery The First Affiliated Hospital of China Medical University Shenyang China

**Keywords:** accelerated aging, cancer, DNA methylation, network analysis, pan‐cancer

## Abstract

Cancers are known to be associated with accelerated aging, but to date, there has been a paucity of systematic and in‐depth studies of the correlation between aging and cancer. DNA methylation (DNAm) profiles can be used as aging markers and utilized to construct aging predictors. In this study, we downloaded 333 paired samples of DNAm, expression and mutation profiles encompassing 11 types of tissues from The Cancer Genome Atlas public access portal. The DNAm aging scores were calculated using the Support Vector Machine regression model. The DNAm aging scores of cancers revealed significant aging acceleration compared to adjacent normal tissues. Aging acceleration‐associated mutation modules and expression modules were identified in 11 types of cancers. In addition, we constructed bipartite networks of mutations and expression, and the differential expression modules related to aging‐associated mutations were selected in 11 types of cancers using the expression quantitative trait locus method. The results of enrichment analyses also identified common functions across cancers and cancer‐specific characteristics of aging acceleration. The aging acceleration interaction network across cancers suggested a core status of thyroid carcinoma and neck squamous cell carcinoma in the aging process. In summary, we have identified correlations between aging and cancers and revealed insights into the biological functions of the modules in aging and cancers.

**Abbreviations**
BLCA: bladder urothelial carcinoma
BP: biological process
BRCA: breast invasive carcinoma
COAD: colon adenocarcinoma
DNAm: DNA methylation
eQTL: expression quantitative trait locus
ESCA: esophageal carcinoma
FDR: false discovery rate
GO: Gene Ontology
GSEA: Gene Set Enrichment Analysis
HNSC: head and neck squamous cell carcinoma
KEGG: Kyoto Encyclopedia of Genes and Genomes
KIRC: kidney clear cell carcinoma
KIRP: kidney papillary cell carcinoma
LASSO: least absolute shrinkage and selection operator
LIHC: liver hepatocellular carcinoma
LOOCV: leave‐one‐out cross validation
LUAD: lung adenocarcinoma
mRMR: minimum redundancy maximum relevance
MSE: mean square error
PRAD: prostate adenocarcinoma
ROC: receiver operating characteristic
SVD: singular value decomposition
SVM: Support Vector Machine
THCA: thyroid carcinoma

Cancers are a major cause of mortality across ethnicity, gender and age groups 1. With regard to the cancer burden expanding due to the growth and aging of the population 2, thorough studies of cancer are increasingly gaining attention. Recent studies have focused on pan‐cancer analyses 3, 4, and a series of studies have revealed that human tumors could be re‐classified based on clustering methods 5. Moreover, systematically studying tumor‐associated biological processes (BPs) and signaling pathways has helped us learn more about similarities of mechanisms and patterns in tumors 6. For instance, several important tumor‐associated signaling pathways have been identified as frequently genetically altered in cancers, such as the cell cycle, Hippo and Myc pathways 6, 7.

The trend of an aging population has resulted in aging becoming a major topic. Aging affects the functional regeneration of tissues, resulting in the accumulation of a degree of malignancy in cells and tissues. It has now been well established that cancers and aging share similar characteristics such as genomic instability, mutations and intercellular signal exchanges 8. Consequently, recent studies have attached great importance to the association between cancers and aging. Identifying age‐associated CpG methylation sites has helped us to understand the fundamental biology of aging and the risk of diseases like cancer 9, and indicated that cancers show significant aging acceleration 10. For instance, research into mutant NOTCH1 clones colonizing the human esophagus with age suggested a complex relationship between aging and cancers 11. Age‐associated DNA methylation changes have been widely reported across multiple tissues and blood 12, so quite a few researchers have emphasized integrating methylation data of multiple tissues to predict age (groups), and this has demonstrated remarkable accuracy 10, 13.

Integrating multi‐omics profiles such as DNA methylation, somatic mutation and expression profiles has provided us with a meaningful and comprehensive study of the BPs involved in cancer development and progression. The elastic net regression model was used to regress age on methylation levels, with 353 aging markers selected 10. To address the association between mutation data and expression profiles, the expression quantitative trait locus (eQTL) method has been proposed 14, 15, and this has been used to identify mutation modules whose alterations were most likely to contribute to abnormal expression of the target genes 16. In recent years, methods based on networks have emerged as powerful tools for studying complex diseases (i.e. cancers) 17, 18. Additionally, further research has studied pan‐cancer from the perspective of dysregulation modules 17 and gained insights into the underlying biological theme of selected gene modules. However, there were still plenty of limitations in systematic studies of pan‐cancer in the context of aging acceleration.

To address this issue, we constructed an aging predictor to discriminate age groups (young versus old) and calculated aging acceleration based on DNA methylation (DNAm) profiles 10. Then, we utilized aging acceleration, mutation profiles and expression profiles in order to revealed insights into the association between cancers and aging. In addition, we identified enriched genes in Kyoto Encyclopedia of Genes and Genomes (KEGG) pathways and Gene Ontology (GO) BP terms 19 in order to explore the biological significance of the identified modules and constructed an aging acceleration interaction network across cancers. In summary, our study has significance for understanding the correlations of cancers and studying the relationship between cancers and aging, as well as contributing to disease diagnosis.

## Results and Discussion

### A brief description of the work‐flow

**Figure 1:**
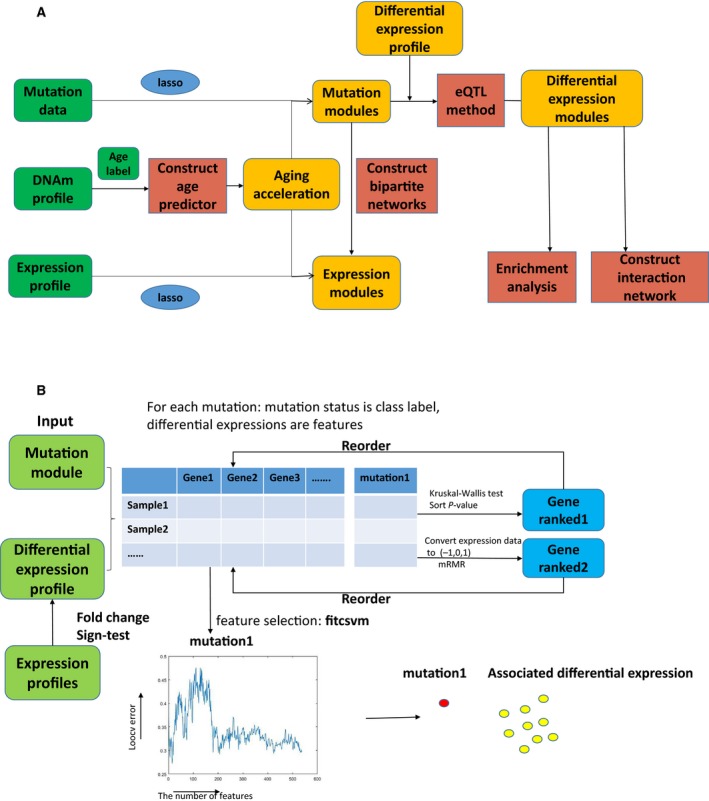
The work flow. (A) The work flow in this study. (B) The work flow for identifying mutation‐associated differential expression modules using the eQTL method.

To explore the potential impact of aging on cancers, DNAm, somatic mutation and expression profiles were used for a series of analyses. The pipeline is shown in Fig. 1A and illustrated as follows (performed in matlab, Beijing, China):

1. the aging predictor was modeled based on the Support Vector Machine (SVM) method using the DNAm profiles of candidate markers, and then the aging acceleration was calculated to test statistical significance of cancers;
2. the least absolute shrinkage and selection operator (LASSO) regression method was utilized to identify the aging acceleration‐associated mutation sets and expression sets in each cancer;
3. the eQTL method was performed to identified the mutation‐associated differential expression module in each cancer.

The eQTL method is introduced in Fig. 1B:

1. for each mutation, the mutation status was the class label, differential expressions (the sign‐test *P*‐value < 0.05, false discovery rate (FDR) < 0.2 and fold change &gt; 2) were candidate features; further, the eQTL method was applied to identify mutation‐associated differential expression modules in the candidate features (matlab);
2. the genes were rearranged by the Kruskal–Wallis test and minimum redundancy maximum relevance (mRMR) method (matlab);
3. leave‐one‐out cross validation (LOOCV) was performed to determine the size of the differential expression module based on the smallest mean error (matlab).

In addition, to explore the functional role of the identified differential expression modules, an enrichment analysis was performed (perl, ActiveState, Vancouver, BC, USA and matlab); and the aging acceleration interaction network across cancers was constructed using the data of mutation‐associated differential expression modules based on discretized K‐S statistics; the details of the analysis are shown in Materials and methods (matlab). The programs and versions of our study are shown in Table S1.

### Modelling the DNAm aging predictor and calculating the age acceleration

To construct a multi‐tissue DNAm aging score predictor, the Kruskal–Wallis test was applied to identify CpGs whose DNAm levels were significantly associated with age. Consequently, 537 CpG sites were observed (*P*‐value < 0.05 and FDR < 0.2) (Table S2). The 537 DNAm sites were considered as candidate epigenetic aging markers and have been utilized to develop the DNAm aging score predictor 9.

**Figure 2:**
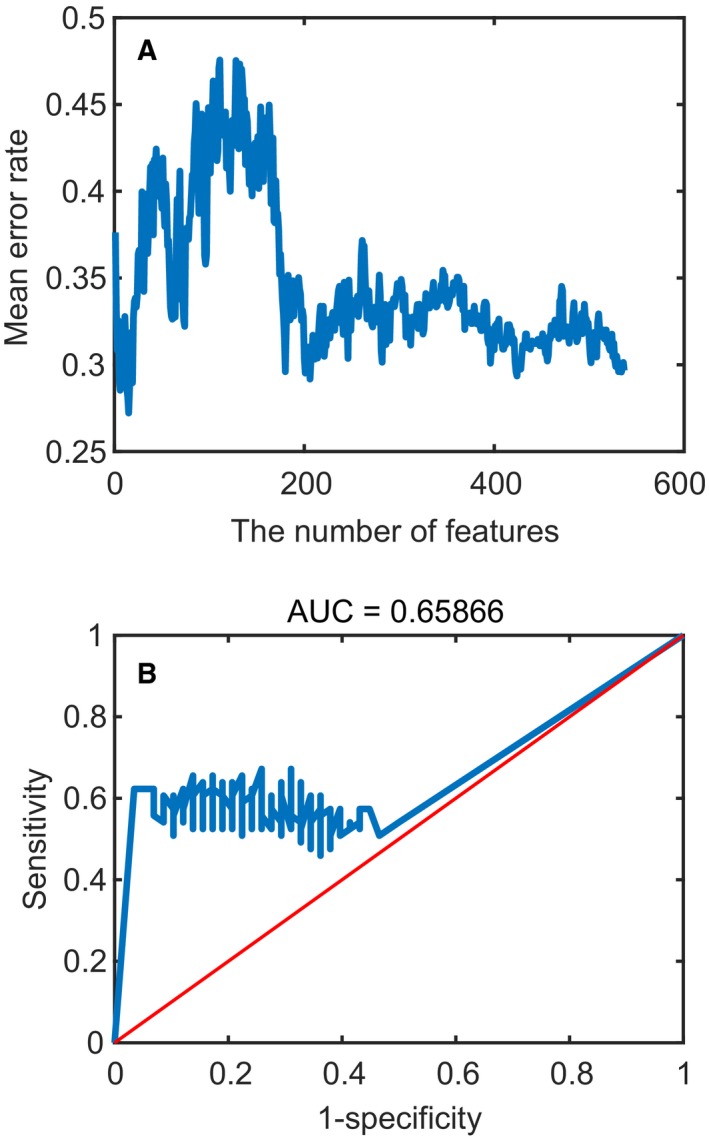
Aging predictor results. (A) The learning curve (mean error rate) of nine‐fold cross‐validation. (B) The ROC curve of test data sets. AUC, area under the ROC curve.

In order to divide 333 samples into young and old groups, the training data sets of DNAm were utilized to train an SVM regression model. Nine‐fold cross‐validation (leaving one type of normal tissues as the temporary test data set every time) was carried out to evaluate the performance of the model and prevent over‐fitting. Ultimately, the optimal model (including 15 aging markers) was selected and the error rate of nine‐fold cross‐validation was 0.2722. The learning curve of the model is shown in Fig. 2A. The test data sets were used to calculate the error rate of the SVM regression model and the receiver operating characteristic (ROC) curve is shown in Fig. 2B. The DNAm values of 15 aging markers were used to evaluate the performance of the trained model and the error rate was 0.2648, which indicated the proper performance of the aging score predictor based on the SVM regression model across tissues. The aging score predictor contained 15 aging markers (Table 1). Many studies have demonstrated that these aging markers are closely related to aging and cancer (https://www.genecards.org/). For instance, *NELL2* was most closely related to aging (*P*‐value = 2.08 × 10^−11^ and FDR = 5.41 × 10^−7^), and studies have shown that this gene plays a role in neural cell growth and differentiation as well as in oncogenesis 20 and is involved in the modulation of mitogen‐activated protein kinase pathways 21. Mitogen‐activated protein kinase pathways are known to play an important part in progression of this cancer 22. Obviously, *NELL2* was closely related to the occurrence of cancer. Further, *SLC9A7* (*P*‐value = 3.44 × 10^−10^ and FDR = 2.98 × 10^−6^) is involved in enhancing cell growth of certain tumors 23 and is associated with multiple neurological syndromes 24.

**Table 1:** Fifteen aging markers

Gene index	Gene symbol	*P*‐value*	FDR	Function
cg06493994	*NELL2*	2.08 × 10^−11^	5.41 × 10^−7^	Plays a role in neural cell growth and differentiation as well as in oncogenesis
cg04084157	*GRPEL1*	8.59 × 10^−11^	1.12 × 10^−6^	Its related pathways are Mitochondrial protein import and Metabolism of proteins
cg19996355	*SLC9A7*	3.44 × 10^−10^	2.98 × 10^−6^	Enhances cell growth of certain breast tumors
cg22736354	*GPR45*	5.21 × 10^−10^	3.39 × 10^−6^	Mediates signaling processes to the interior of the cell via activation of heterotrimeric G proteins
cg12373771	*C9orf72*	8.80 × 10^−10^	4.57 × 10^−6^	Plays a role within the hematopoietic system in restricting inflammation and the development of autoimmunity
cg20300246	*CPNE3*	1.38 × 10^−9^	5.40 × 10^−6^	Plays a role in ERBB2‐mediated tumor cell migration in response to growth factor heregulin stimulation
cg17497271	*CARD4*	1.45 × 10^−9^	5.40 × 10^−6^	Is involved in apoptotic signaling, LRRs participate in protein–protein interactions
cg07850604	*FOSL2*	5.69 × 10^−9^	1.85 × 10^−5^	Is implicated as regulator of cell proliferation, differentiation, and transformation
cg23739862	*NRP2*	9.30 × 10^−9^	2.69 × 10^−5^	Plays a role in cardiovascular development, axon guidance, and tumorigenesis
cg02331561	*TSC2*	1.63 × 10^−8^	4.25 × 10^−5^	Is a tumor suppressor and is able to stimulate specific GTPases
cg20051033	*MAGEH1*	2.06 × 10^−8^	4.83 × 10^−5^	Is associated with apoptosis, cell cycle arrest, growth inhibition or cell differentiation
cg18809289	*C16orf63*	2.23 × 10^−8^	4.83 × 10^−5^	Is required for the recruitment of PLK1 to centrosomes and S phase progression
cg18267374	*UNQ9433*	2.74 × 10^−8^	5.47 × 10^−5^	Ligand for receptor tyrosine kinase LTK and perhaps receptor tyrosine kinase ALK
cg16778903	*PHB2*	3.45 × 10^−8^	6.40 × 10^−5^	Is involved in regulating mitochondrial respiration activity and in aging
cg17861230	*JAKMIP2*	5.64 × 10^−8^	9.77 × 10^−5^	A component of the Golgi matrix

*Calculated by the Kruskal–Wallis test. *Calculated by the Kruskal–Wallis test. LRRs, leucine‐rich repeats; PLK1, polo like kinase 1.

**Figure 3:**
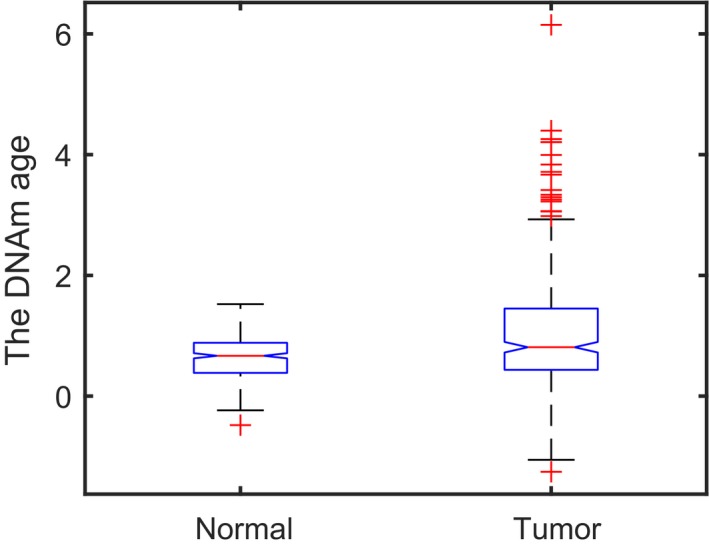
The results of the Kruskal–Wallis test for DNAm age between tumor and normal samples.

After this, the DNAm profiles of adjacent normal tissues and cancers were put into the aging predictor to calculate the respective DNAm aging scores (Table S3). The results demonstrated that the DNAm aging scores of cancers showed significant aging acceleration compared to the DNAm aging scores of adjacent normal tissues (Kruskal–Wallis test: *P*‐value = 9.7924 × 10^−7^; Fig. 3), and the median DNAm aging score of cancers was 0.1415 higher than the median DNAm aging score of adjacent normal tissues and the mean DNAm aging score of cancers was 0.3828 higher than the mean DNAm aging score of adjacent normal tissues. Indeed, previous research has demonstrated significant aging acceleration in multiple tissues, brain regions and the blood 10, 12.

### Identifying aging acceleration‐associated mutation and expression modules

**Figure 4:**
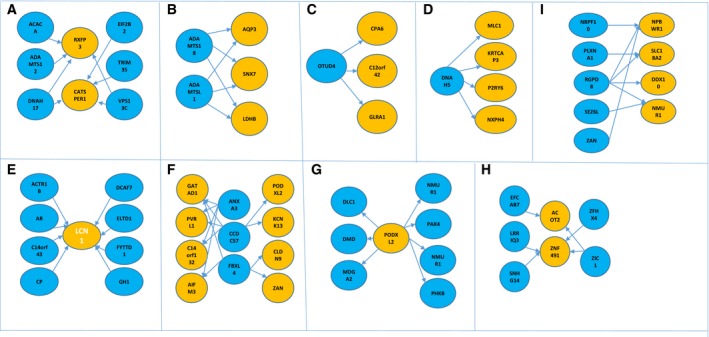
The bipartite networks of aging acceleration‐associated mutation modules and expression modules. (A) BLCA; (B) COAD; (C) ESCA; (D) HNSC; (E) KIRP; (F) LIHC; (G) LUAD; (H) PRAD; (I) THCA. The blue circles represent mutations and the yellow circles represent expression.

To deepen insight into the aging acceleration across cancers, the LASSO regression method was used to identify an aging acceleration‐associated mutation set and expression set in each cancer, where aging acceleration was calculated by the DNAm aging score of cancers minus the paired DNAm aging score of adjacent normal tissues. Five‐fold cross‐validation was utilized to select the optimal model with the smallest mean square error (MSE). These identified sets are shown in Tables [Link], [Link]. The Kruskal–Wallis test was performed to identify edges between mutations and expression and to construct bipartite networks (shown in Fig. 4). Bipartite networks were constructed in bladder urothelial carcinoma (BLCA), colon adenocarcinoma (COAD), esophageal carcinoma (ESCA), head and neck squamous cell carcinoma (HNSC), kidney papillary cell carcinoma (KIRP), liver hepatocellular carcinoma (LIHC), lung adenocarcinoma (LUAD), prostate adenocarcinoma (PRAD) and thyroid carcinoma (THCA). It was noteworthy that the most significant mutation–expression connection was *ELTD1*–*LCN1* in KIRP (the Kruskal–Wallis test, *P*‐value: 6.9 × 10^−4^, FDR: 1.3 × 10^−3^). *ELTD1* is involved in G protein‐coupled receptor activity 25 and transmembrane signaling receptor activity. In addition, studies have shown that when the expression of *ELTD1* was silenced, tumor invasiveness was significantly reduced 26. *LCN1* is an important lipocalin that plays a major role in inflammation and cancer 27. Obviously, the bipartite networks revealed key relations between aging and cancers.

### Functional analysis across cancers based on aging acceleration

**Figure 5:**
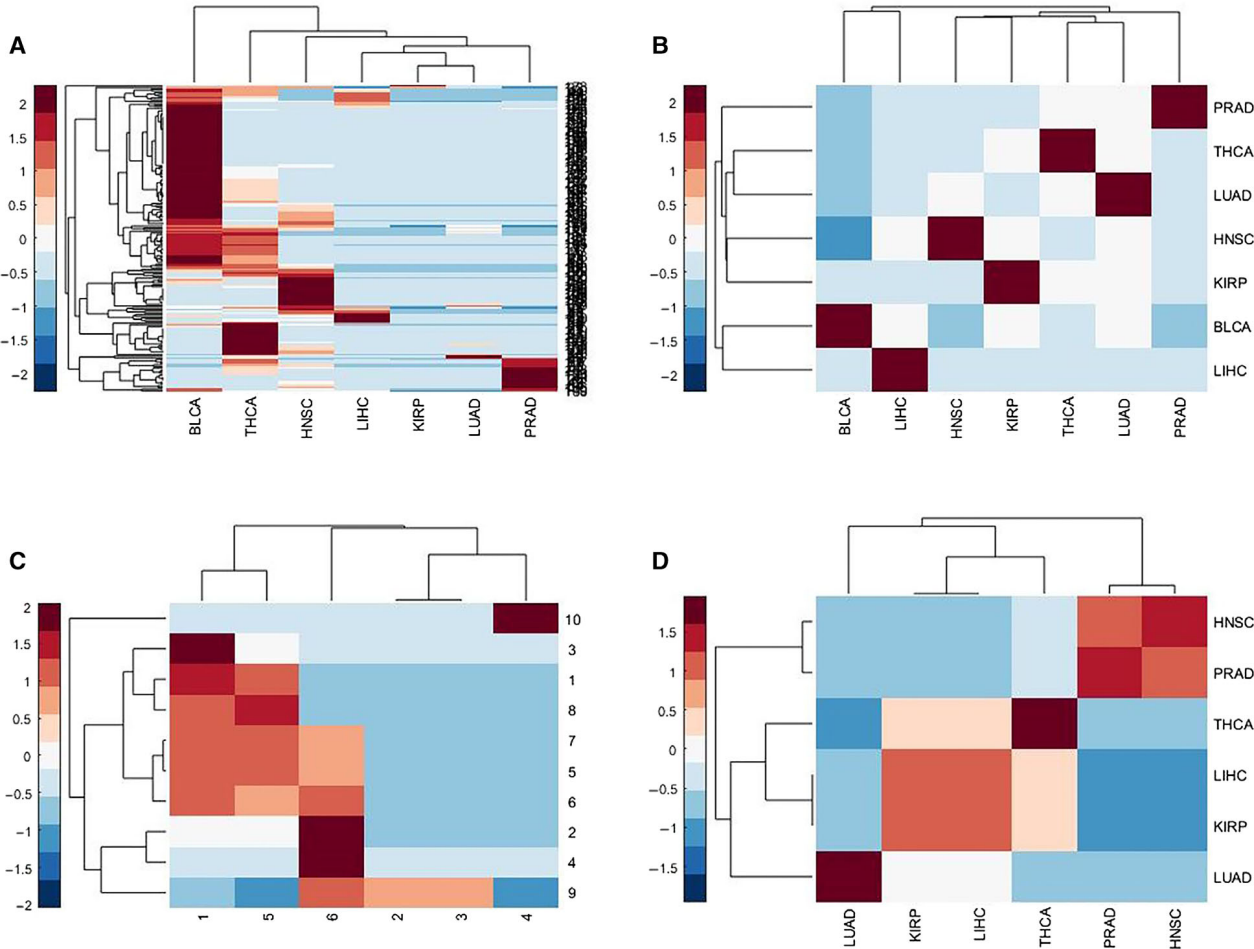
Enrichment results. (A) Heat map of GO BP terms with statistical significance. (B) Heat map of the correlation coefficient of cancers based on (1 − FDR) of GO BP terms. (C) Heat map of KEGG pathways with statistical significance. (D) Heat map of the correlation coefficient of cancers based on (1 − FDR) of KEGG pathways.

To gain a deeper understanding of the biological functions of mutation‐related differential expression modules, the eQTL method was used to identify differential expression modules that were affected by aging acceleration‐associated mutations, and the hypergeometric test was performed to identify enriched genes in KEGG pathways and GO BP terms (Materials and methods). The differential expression modules were identified in 11 types of cancers and were used to explore related biological functions. The results of the enrichment analysis showed that mutation‐related differential expression modules in HNSC, KIRP, LIHC, LUAD, PRAD and THCA were significantly enriched into KEGG pathways (Table S6) and mutation‐related differential expression modules in HNSC, KIRP, LIHC, LUAD, PRAD and THCA were significantly enriched to GO BP terms (Table S7). To clearly show the significance of certain KEGG pathways or GO BP terms in different cancers, heat maps of KEGG pathways or GO BP terms were plotted (Fig. 5A,C). It could be intuitively observed that different cancers shared the same pathways or terms, which meant that different cancers had similarities in pathways. For instance, the differential expression modules in BLCA, HNSC, KIRP and THCA were significantly enriched for the GO BP term ‘cell–cell signaling’ (GO: 0007267), which is involved in any process that mediates the transfer of information from one cell to another and always carried out in the living body. Cells could recognize various signals present in the surrounding environment when the body is faced with aging or cancers and transform them into various molecular changes in the cell, thereby changing or adjusting certain behaviors in the cell, such as metabolic processes, affecting the growth rate of cells, and even inducing cell death. Recent studies have shown that redox signaling is a key component of cellular signaling pathways, in which individual components of the Srx–Prx system play important roles in carcinogenesis by modulating the cell signaling pathways involved in cell proliferation, migration and metastasis 28. Moreover, differential expression modules in various cancers were enriched in the GO BP terms ‘regulation of synapse organization’ (GO: 0050807) (BLCA, LIHC, THCA) 29 and ‘regulation of hormone levels’ (GO: 0010817) (BLCA, HNSC, THCA) 30, which have been proven to be closely related to aging. The KEGG pathway ‘neuroactive ligand receptor interaction’ (KIRP, LIHC, THCA) was closely associated with cancer 31.

The results of the enrichment analyses also showed the characteristics of certain cancers. For instance, the differential expression module in BLCA was enriched in GO BP term ‘response to nitrogen compound’ (GO: 1901698) (*P*‐value = 0.0037 and FDR = 0.171) 32, and the differential expression module in THCA was enriched to GO BP term ‘ovulation cycle’ (GO: 0010817) (*P*‐value = 0.0014 and FDR = 0.1814). At present, studies have shown that abnormal thyroid function affects the level of reproductive hormones, thus affecting women's ovulation cycle 33. More specifically, we could observe that the most significant GO BP term was ‘modulation of synaptic transmission’ (GO: 0050804) in BLCA (*P* = 9.0041 × 10^−10^ and FDR = 3.99 × 10^−6^) 34. Differential expression modules in different cancers were enriched in pathways associated with neural signals such as ‘neuropeptide signaling pathway’, ‘neuron development’, ‘positive regulation of synaptic transmission, glutamatergic’, and so on. The results were consistent with the current studies that the nervous system played an important regulatory role in the process of aging and cancer 35, 36.

In order to reveal insights into associations of pathways between cancers and the whole pattern across cancers, the pathways and terms were selected with a FDR < 0.2 (through the Gene Set Enrichment Analysis (GSEA) platform 37), and The correlation coefficient between cancers was calculated based on the (1 − FDR) values of pathways or terms. The heat maps are shown in Fig. 5B,D. According to the cancer‐related heat map based on KEGG pathways, it could be observed that the KEGG pathways of HNSC and PRAD had a high similarity and the correlation coefficient was 0.85, indicating the similarity in the pathway changes between HNSC and PRAD during carcinogenesis. The correlation coefficients between KIRP and THCA, and LIHC and THCA were both 0.37.

### Constructing aging acceleration interaction network across cancers

**Figure 6:**
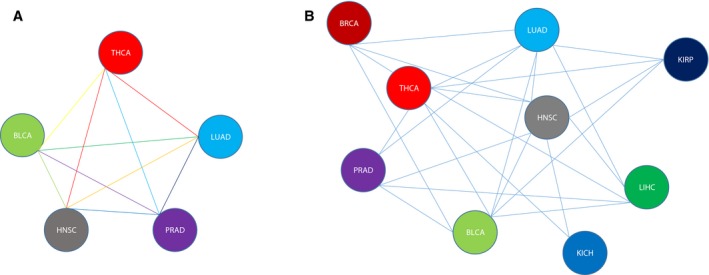
The aging acceleration interaction network across cancers. (A) constructed by top 10 edges; (B) constructed by edges whose values are larger than 100.

To address similarities and associations of the aging process across cancers, an aging acceleration interaction network across cancers was constructed using Kolmogorov–Smirnov statistics (Table 2). The top 10 edges were selected (Fig. 6A). The network contained BLCA, HNSC, LUAD, PRAD and THCA, where the top edge connected HNSC and THCA with a score of 901.38. The result suggested that HNSC and THCA were related to each other in the process of aging and carcinogenesis, consistent with a study finding that the expression of SDCBP was upregulated in both HNSC and THCA 38. In order to connect more cancer types, the edges with score > 100 were selected (Fig. 6B). The network included breast invasive carcinoma (BRCA), HNSC, kidney clear cell carcinoma (KIRC), KIRP, LIHC, LUAD, PRAD and THCA. The network contained 25 edges and the highest degree of cancers were HNSC and THCA with a degree of 8, which suggested a core position for HNSC and THCA and that they may regulate other cancers in aging processes 39, 40. It was worth noting that the edges of THCA and BRCA 41, and THCA and PRAD 42 were high (Table 2), which indicated the association between cancers. Studies have shown that dysfunction of the thyroid led to imbalance of sex hormone levels 43, 44, resulting in cancer of organs regulated by sex hormones such as the breast and the prostate. In summary, our analyses were consistent with previous studies and were credible.

**Table 2:** Aging acceleration interaction network across cancers.

Cancer	BRCA	COAD	ESCA	HNSC	KIRC	KIRP	LIHC	LUAD	PRAD	THCA
BLCA	128.94	57.068	31.511	613.09*	70.477	127.4	189.33	491.54*	287.68*	627.91*
BRCA	–	20.403	11.038	199.4	20.392	39.677	59.038	165.64	91.3	191.13
COAD	–	–	5.4002	76.899	11.922	18.835	26.894	64.872	38.252	86.36
ESCA	–	–	–	48.617	6.0181	10.78	15.982	40.428	23.236	50
HNSC	–	–	–	–	115.49	182.2	265.26	666.26*	379.87*	901.38*
KIRC	–	–	–	–	–	22.277	33.443	94.94	51.522	106.57
KIRP	–	–	–	–	–	–	56.655	153.34	86.397	187.9
LIHC	–	–	–	–	–	–	–	219.54	124.62	277.35
LUAD	–	–	–	–	–	–	–	–	329.67*	733.63*
PRAD	–	–	–	–	–	–	–	–	–	418.88*

*Number representing top 10 edges.

## Conclusion

In summary, 15 DNAm markers were selected from 537 candidate markers based on cross‐validation; as a result, they were considered as aging markers to construct the DNAm aging predictor. The results indicated that the DNAm aging scores of cancers showed significant aging acceleration compared to the adjacent normal tissues, which was consistent with previous studies 10, 12. Aging acceleration‐associated mutation sets and expression sets were identified and bipartite networks were constructed. Further, the functional role of significant edges was also demonstrated by previous studies 25, 26. In addition, differential expression modules related to aging acceleration‐associated mutation were identified by the eQTL method. An enrichment analysis was performed to provide insight into the biological functions of these identified modules. The results suggested that different types of cancers shared the same GO BP terms or KEGG pathways such as cell–cell signaling and pathways associated with neural signals. Moreover, the pathway characteristics of certain cancers were also observed. Some pathways were highly related across cancers. Aging acceleration interaction network across cancers suggested the core position of THCA and HNSC in aging and cancers. The correlation between THCA and BRCA was high, which implied a relationship of THCA and BRCA in cancers and the regulation of thyroid on sex hormones. Generally, our computational processes revealed the association between aging and cancers.

## Materials and methods

### Samples and data set description

The paired DNA methylation profiles (333 × 25 978), expression profiles (333 × 14 530), mutation profiles (333 × 15 713) and corresponding clinical data (333 × 1) of both adjacent normal tissues and cancers were downloaded from The Cancer Genome Atlas public access portal (through the xena platform https://xenabrowser.net/hub/). The data were composed of a total of 333 samples, encompassing 11 tissues (Table 3): BLCA, BRCA, COAD, ESCA, HNSC, KIRC, KIRP, LIHC, LUAD, PRAD and THCA. The tissues whose number of samples was greater than 10 were selected.

**Table 3:** Description of data. The tissues marked with an asterisk represent test data sets, the remaining tissues represent training data sets.

Tissues	No. of samples	No. of young samples	No. of old samples
BLCA	17	5	12
BRCA*	78	46	32
COAD	16	4	12
ESCA	11	4	7
HNSC	20	5	15
KIRC	24	6	18
KIRP	23	9	14
LIHC*	41	15	26
LUAD	18	8	10
PRAD	35	12	23
THCA	50	37	13

The process of data standardization was shown as follows. For each tissue, the singular value decomposition (SVD) method 45 was performed on DNA methylation data in order to offset variations between different tissues, and each gene was normalized using the *z*‐score method. More specifically, for the DNAm data of cancer and the adjacent normal tissue (all samples corresponding to each gene in each tissue), *z*‐score normalization was performed based on the mean and standard deviation of the adjacent normal tissue (all samples corresponding to each gene in each tissue). Then, for the standardized data of the cancer and the adjacent normal tissue, SVD normalization was performed based on the top three principal component of the adjacent normal tissue. Ultimately, for the standardized data of the cancer and the adjacent normal tissue, *z*‐score normalization was performed based on the mean and standard deviation of the adjacent normal tissue. The median normalization was performed on gene expression data of cancers and adjacent normal tissues. The mutation matrix was a 0 or 1 matrix: if a gene occurred with a non‐synonymous mutation in a sample, the value was set to 1; otherwise, it was set to 0.

### Modeling a multi‐tissue DNAm aging predictor and aging acceleration

In order to model the aging predictor, all the normal samples were divided into two parts. The choice of training data sets was selected by the following criteria:

1. the ratio of the number of samples in the training data set to that of the test data set was about 2 : 1;
2. the ratio of young samples (age ≤ 60) to old samples (age &gt; 60) in the training data set was approximated to that in the test data set;
3. the training data sets should represent a wide spectrum of tissues and cell types. According to the criteria, the training data set (214 × 25 978) encompassed nine types of tissues: BLCA, COCA, ESCA, HNSC, KIRC, KIRP, LUAD, PRAD and THCA. The test data set (119 × 25 978) encompassed two types of tissues: BRCA and LIHC (Table 3).

The clinical age was labeled as 1 if the age was greater than 60, or else labeled as 0. Then, the Kruskal–Wallis test was applied to the training data set of DNAm data and the corresponding age labels. These DNAm sites were identified as candidate aging markers after Benjamini–Hochberg FDR adjustment. The threshold was *P* value < 0.05 and FDR < 0.2. Next, these aging markers were utilized to construct a multi‐tissue DNAm aging predictor. The SVM regression model was applied to classify the samples into young (aging score ≤ 0.5) and old (aging score > 0.5) groups. The fitrsvm function was called in matlab. The input parameters were DNAm values of training data sets and age labels (0 or 1) and the output parameter was the trained model. The DNAm values of test data sets were put into the trained model and the regression values of aging were obtained. The continuous regression values were divided into 1 and 0 (aging score > 0.5 and aging score ≤ 0.5). The average error rate of the classification was calculated, namely, the mean of false negative and false positive. The process of constructing the aging predictor was as follows:

1. The nine‐fold cross‐validation was used to select the optimal model, evaluating the performance of the model and preventing overfitting. Each time, one tissue was selected from the nine tissues in the training data set as a temporary test data set and the remaining eight tissues as temporary training data sets. The temporary training data sets were used to train the SVM model and the temporary test data set was used to calculate the average error rate of the classification. This process was cycled nine times. Ultimately, the model with the lowest average error rate was chosen. The identified features were considered as aging markers.
2. The selected aging markers of the whole training data were used to train the SVM model and construct an aging predictor; then, test data of corresponding aging markers were input into the trained SVM model to discriminate age groups; afterwards, the average error rate of the classification was calculated. The DNAm profiles of adjacent normal tissues and cancers were put into the trained aging predictor to calculate DNAm aging scores, respectively. Then, the age acceleration was calculated through DNAm aging scores of cancers subtracting adjacent normal tissues. Finally, the Kruskal–Wallis test was applied to test the significance of aging acceleration.

### Identifying aging acceleration associated mutation module and expression module in each cancer

In each cancer, LASSO regression was used to identify aging acceleration‐associated mutation sets and expression sets. The LASSO regression function was called in matlab (R2015b). The input parameters were: mutation matrix of each cancer and aging acceleration vector. The ‘alpha’ was set to 1, the ‘cross‐validation’ was set to 5. The output parameter B was fitted coefficients, a *p*‐by‐*L* matrix, where *p* was the number of predictors (columns) in *X*, and *L* was the number of lambda values. The FitInfo was a structure, where the MSE could be used to evaluate the performance of the model. The model with the smallest MSE and minimal complexity was selected and mutations in the model whose coefficients were not zero were chosen. These mutations constituted the aging acceleration‐associated mutation set. The expression data of cancers minus the expression data of adjacent normal tissues and the matrix *D* were obtained. The same method was used to identify expression sets related to aging acceleration.

To construct bipartite networks, the Kruskal–Wallis test was applied to aging acceleration‐associated mutation modules and expression modules. Then *P*‐values were obtained and FDR adjustment performed and the threshold was *P*‐value < 0.05 and FDR < 0.2. Research has shown that different FDRs can be applied to identify mRNA in different types of cancers, such as FDR from < 0.1 to < 0.5 18. In this work, FDR < 0.2 was used in each cancer in order to unify standards. The significant mutation–expression pairs were visualized in bipartite networks.

### Identifying differential expression modules related to aging acceleration associated mutations

To identify mutation‐associated differential expression modules in each cancer, the eQTL method was applied (Fig. 1B) 15. Generally, eQTL analysis was used to identify the genotypes of genomic locations that significantly affect gene expression 46. However, we could also consider that one mutation could affect one or multiple expressions. In other words, the relationship between mutation and gene expression was mutual 15. Different from traditional eQTL methods, the eQTL we used was an information theory‐based machine learning method.

First, differential expression genes were selected as candidate genes, which complied with the following criteria:

1. the sign‐test was applied to the expression of cancers and adjacent normal tissues and FDR adjustment performed; the threshold was *P*‐value < 0.05 and FDR < 0.2;
2. the fold‐change was greater than 2.

Next, the Kruskal–Wallis test was applied to differentially expressed genes and each mutation and gene was sorted in ascending order by *P* value. For the data of sorted genes, ternary discretization was executed (by mean ± SD/2: 1, 0, −1).

Further, in order to identify mutation‐associated expression modules in candidate genes by solving the problem of the minimum coverage set, where the mRMR searching algorithm was applied to rank the expressions according to their relevance both to the mutation and to the redundancy among the expressions. The mRMR function was defined as

(1) $${\text{max}}_{g_{j}\in G}\left(R_{j}-D_{j}\right)$$

where *R* represents the relevance of a gene *g* in *G* and the mutation label *l* and was defined as

(2) $$R={\text{max}}_{G}\frac{1}{\left|G\right|}\sum_{g_{i}\in G}I\left(g_{i};l\right)$$

*D* represents the redundancy of a genes and was defined as

(3) $$D={\text{min}}_{G}\frac{1}{{\left|G\right|}^{2}}\sum_{g_{i},g_{j}\in G}I\left(g_{i};g_{j}\right)$$

In the case of discrete values of expressions, the mutual information is defined as:

(4) $$I\left(x;y\right)=\sum_{y\in Y}\sum_{x\in X}p\left(x,y\right)*\log\left(\frac{P(x,y)}{p(x)p(y)}\right)$$

Finally, LOOCV 47 was utilized to determine the size of the differential expression module.

### The enrichment analysis based on the hypergeometric test

To understand the biological function of differential expression modules, the enrichment analysis was applied to the analysis of GO BP terms and KEGG pathways 19. We downloaded the information for GO terms (including all GO gene sets), GO BP and KEGG pathways (http://software.broadinstitute.org/gsea/downloads.jsp, version 6.2), and the latest study has shown that GSEA analyses provided biologically meaningful insights in gene lists with an FDR < 0.25 37). The hypergeometric distribution was a discrete probability distribution, which was performed to estimate the enrichment of these selected markers compared to known terms or pathways. The formula of the hypergeometric test was:

(5) $$P\left(X\geq x\right)=1-\sum_{k=0}^{x-1}\frac{C_{M}^{k}*C_{N-M}^{n-k}}{C_{N}^{n}}$$

where *N* is the total gene number of the gene sets, *M* is the number of known gene sets (i.e. GO terms or KEGG pathways), *n* is the number of the candidate genes that we identified and *k* is the number of common entries between them. *P* was the enrichment statistical significance of the test. *P*‐value was justified based on Benjamini–Hochberg FDR and the threshold was *P*‐value < 0.05 and FDR < 0.2 37.

The corr function was called in matlab to calculate the correlation coefficient between pathways of different cancers. These pathways were picked out if some differential expression modules were significantly enriched on the pathways. Eventually, an 11 × *N* FDR matrix was obtained, where *N* was the number of enriched pathways.

### Constructing an aging acceleration interaction network across cancers

In order to study the potential relationship between the identified differential expression modules and aging across cancers, we constructed an aging acceleration interaction network across cancers. The sample was considered to be an aging accelerated sample if the value of aging acceleration was greater than 0, else it was considered to be a non‐aging‐accelerated sample. For each pair of cancers, the accumulated Kolmogorov–Smirnov (K‐S) statistics 48 of every gene pair in aging‐accelerated samples and non‐aging‐accelerated samples were calculated. The formula was

(6) KS=sup|F1-F2|

where F1 and F2 represented cumulative probability distributions of the same type of samples (aging accelerated or non‐aging accelerated) in the two cancers. We calculated the cumulative K‐S statistics of aging‐accelerated samples and non‐aging‐accelerated samples for each cancer. Then, the absolute value of cumulative K‐S statistic difference between aging‐accelerated samples and non‐aging‐accelerated samples was calculated. Since the number of gene pairs differed from different cancers, the cumulative value should be divided by the number of gene pairs. The formula was

(7) $$\text{similarity}=\frac{\sum_{i=1}^{N_{1}*N_{2}}{\text{KS}}_{\mathrm{accelerated}}-{\text{KS}}_{\mathrm{non}-\mathrm{accelerated}}}{\ln\left(N_{1}\right)+\ln\left(N_{2}\right)}$$

where *N*_1_ indicates the number of genes in the differential expression module of cancer 1, *N*_2_ indicates the number of genes in the differential expression module of cancer 2, KS_accelerated_ indicates the K‐S statistics of aging‐accelerated samples and KS_non‐accelerated_ indicated the K‐S statistics of non‐aging accelerated samples. The normalized value was used as the length of the edge (similarity) between cancer pairs in the interaction network.

## Conflict of interest

The authors declare no conflict of interest.

## Author contributions

XX, MZ, HY and YW performed the algorithm and analyzed the data; XX, SL and YW wrote the manuscript; XS, and YW designed and sponsored the study. All authors read and approved the manuscript.

## Supporting information


**Table S1.** The programs and versions of our study.

**Table S2.** 537 aging marker candidates.

**Table S3.** DNAm age of cancers and adjacent normal tissues and aging acceleration.

**Table S4.** Aging acceleration associated mutation modules across cancers.

**Table S5.** Aging acceleration associated expression modules across cancers.

**Table S6.** The significant KEGG pathways.

**Table S7.** The significant GO BP terms.

**Data S1.** The programs of this work (summarized in Table S1).

## Data Availability

The data supporting the results of this article are included and cited within the article and its additional files.
