# Supplementary material for: Network analysis of aging acceleration reveals systematic properties of 11 types of cancers
Source: FEBS Open Bio. 2019 Jun 24;9(7):1292–304. doi: 10.1002/2211-5463.12679 (PMC6609580; doi:10.1002/2211-5463.12679)
Supplement: Supplementary file 1 — Table S1. The programs and versions of our study. [file FEB4-9-1292-s001.doc]

**Table S1: the programs and versions of our study**

| Task 1 | step1_data_normalization.m (MATLAB)  step1_modeling_age_predictor.m (MATLAB)  roc1.m(MATLAB) |
| --- | --- |
| Task 2 | step2_identity_ex_set__lasso_5.m (MATLAB)  step2_identity_mutation_set__lasso_5.m (MATLAB)  step2_bipartite_networks.m (MATLAB) |
| Task 3 | loginformation1.m (MATLAB)  mrmr3.m (MATLAB)  step3_associate_mutation_expression.m (MATLAB) |
| Task 4 | tempbp2.pl (Perl)  c2.cp.kegg.v6.2.symbols.gmt (GSEA)  c5.bp.v6.2.symbols.gmt (GSEA)  ex_bp.m (MATLAB)  ex_kegg.m (MATLAB)  log_hypergeometric.m (MATLAB)  step4_enrichment_analysis.m (MATLAB) |
| Task 5 | step5_ks_similarity.m (MATLAB) |
